# Mechanical Behaviour of Pin-Reinforced Foam Core Sandwich Panels Subjected to Low Impact Loading

**DOI:** 10.3390/polym13213627

**Published:** 2021-10-21

**Authors:** Ali Farokhi Nejad, Seyed Saeid Rahimian Koloor, Syed Mohd Saiful Azwan Syed Hamzah, Mohd Yazid Yahya

**Affiliations:** 1 Department of Mechanical and Aerospace Engineering, Politecnico di Torino, 10129 Turin, Italy; ali.farokhi@polito.it; 2 Department of Solid Mechanics, AMICI R&D Group, Tehran 1474585745, Iran; 3 Institute for Nanomaterials, Advanced Technologies and Innovation (CXI), Technical University of Liberec (TUL), Studentska 2, 461 17 Liberec, Czech Republic; 4 Department of Aerospace Engineering, Faculty of Engineering, Universiti Putra Malaysia, Serdang 43400, Malaysia; 5 Faculty of Engineering, School of Mechanical Engineering, University Technology Malaysia, Johor Bahru 81310, Malaysia; smsazwan2@live.utm.my or; 6 Centre for Advanced Composite Materials (CACM), Universiti Teknologi Malaysia, Johor Bahru 81310, Malaysia

**Keywords:** composite panel, pin-reinforced sandwich panel, low impact loading, finite element model, energy absorption

## Abstract

As a light structure, composite sandwich panels are distinguished by their significant bending stiffness that is rapidly used in the manufacture of aircraft bodies. This study focuses on the mechanical behaviour of through-thickness polymer, pin-reinforced foam core sandwich panels subjected to indentation and low impact loading. Experimental and computational approaches are used to study the global and internal behaviour of the sandwich panel. The samples for experimental testing were made from glass/polyester laminates as the face sheets and polyurethane foam as the foam core. To further reinforce the samples against bending, different sizes of polymeric pins were implemented on the sandwich panels. The sandwich panel was fabricated using the vacuum infusion process. Using the experimental data, a finite element model of the sample was generated in LS-DYNA software, and the effect of pin size and loading rate were examined. Results of the simulation were validated through a proper prediction compared to the test data. The results of the study show that using polymeric pins, the flexural strength of the panel significantly increased under impact loading. In addition, the impact resistance of the pin-reinforced foam core panel increased up to 20%. Moreover, the size of pins has a significant influence on the flexural behaviour while the sample was under a moderate strain rate. To design an optimum pin-reinforced sandwich panel a “design of experiment model” was generated to predict energy absorption and the maximum peak load of proposed sandwich panels. The best design of the panel is recommended with 1.8 mm face sheet thickness and 5 mm pins diameter.

## 1. Introduction

The potential of weight saving while maintaining structural integrity and stability is the driving force to use composite materials and structures in advanced industries [1,2]. Composite sandwich panels are widely used in industries such as aeronautics, railways, marine and civil engineering due to their high specific strength-to-weight ratio, lightweight properties, acoustic damping, high impact resistance, and high thermal insulation [3,4]. Generally, sandwich panels are made of stiff and rigid face sheets and lightweight and thick core materials. The face sheets are made of metallic sheets, polymer sheets, or fibre-reinforced polymer (FRP) composites. The core materials include low-density polymeric foam, metallic foam, honeycomb, corrugated cores, and balsa [5,6,7,8]. The main purpose of using core materials is to increase the second moment of inertia to increase the bending moment [7]. Using lightweight and strength core materials reduces the total structure’s weight that is important in the aeronautic industry.

The resin infusion fabrication method reduces the production costs compared to using honeycomb cores [3,9]. In addition, sandwich panels have higher specific stiffness compared with traditional composite laminates. Using closed-cell foam as the core material can moderate moisture absorption that is a current issue for FRP composite laminates. However, foam cores are weak under localized impact and the structural stiffness can be degraded by face sheet rupture or shear cracking in the bonding region [10,11]. Therefore, the strength of a sandwich structure mostly depends on the face sheet stiffness. Moreover, the bonding between the core and face sheet is a crucial point to use the maximum capacity of the structure stiffness against impact loading. Recently, several studies have been carried out to increase the bonding properties of sandwich structures. Different methods such as tufting, orthogonal weaving, stitching, and Z-pinning were proposed to increase the bonding strength between the core and face sheets [12,13,14,15,16].

Some research has proposed injected reinforced pins to increase the mechanical strength in sandwich structures with foam cores. This method is much simpler and less expensive than other previously proposed methods. The injected resin pins inside the core increase the bending stiffness of the core, to avoid crack initiation and holding the integrity of the foam [9]. The low-cost production, ease of fabrication, and better bonding compared to other kinds of pinned sandwich panels make PFCS a good candidate for sandwich panels subjected to low impact loading.

The main application of composite sandwich panels is in structures that are under low or high impact loading regarding lightweight design. In other words, composite sandwich panels should be designed for impact-resistant applications [17]. Therefore, understanding the dynamic behaviour and responses of a composite structure in the form of sandwich panels, and the prediction of mechanical strength is important to generate an optimum sandwich panel. Some experimental studies have been conducted to investigate the sandwich panel under different types of loadings [11,18,19]. Several failure modes depending on the loading type have been reported [20]. Moreover, different boundary conditions such as simply support and fully clamped were studied. Some analytical models were established to evaluate the damage and failure of sandwich panels. To observe the damaged location, failure modes and permanent deflection non-destructive methods don’t have enough accuracy and the sample should be cut for internal fractographic analysis. Hence some additional damage due to the cutting process can be created on the sample [21]. Numerical models and especially finite element model is a reliable tool to evaluate the structural integrity of the sandwich panel. Damage initiation, damage propagation, and failure are three stages of damage [17,22,23] that can be observed in a sandwich panel under low impact loading. To evaluate the foam core sandwich (FCS) structures many FE models have been reported by previous research [19,24,25]. Recently some numerical models were proposed to evaluate pinned sandwich structures and hybrid sandwich panels [26,27,28] however, although less attention has been paid to generate a three-dimensional (3D) FE model of pin-reinforced foam core sandwich panel (PFCS). Moreover, the FE model of PFCS can help predict different layer configurations and find the optimum structure against low-impact loading.

The design of experiments (DOE) method is a well-known optimization technique to design a set of experiments that requires a minimal number of runs to be performed and still be able to obtain all the necessary information. The best design of PFCS requires categorizing the main design variables and finding their possible interactions, which cannot be simply assessed by conventional means. It can define the factor levels that will simultaneously satisfy a set of preferred specifications [29]. Applying the DOE technique through computational design helps to reduce the number of experiments and find the effectiveness of each parameter in the design process [30].

In this study, a set of FCS and PFCS sandwich panels with the same geometry and different weight and various diameters of pin-reinforced were fabricated. The indentation tests with various applied loads were performed. A FE model, based on the experimental test was generated and validated through the experiment. The proposed FE model was used for carrying out the DOE optimization. To characterize the significant parameters of PFCS structure under low impact loading, a multi-objective optimization response surface method (RSM) DOE approach was performed and the best design is recognized. After finishing the design matrix the responses of the DOE method were analyzed by analysis of variance (ANOVA). The verified statistical model leads to present empirical model to predict the Energy absorption and maximum peak load as the main outcome of this study.

## 2. Material and Experiment

**Figure 1:**
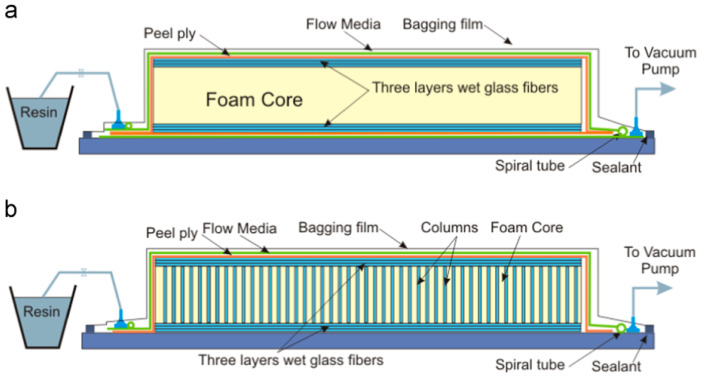
Schematic view of fabrication process of (**a**) (FCS) and (**b**) PFCS panels [3].

Three layers of random chop strand mat glass fibres (produced by Wee Tee Tong Chemicals Pte Ltd., Singapore) with a thickness of 0.33 mm per layer were used for the top and bottom of the sandwich panel. Polyurethane foam core specimens with dimensions 50 × 50 × 11.5 mm and a density of 139.1 kg/m^3^ were used. In this study, the core foams are generated in four categories (FCS, PFCS 1 mm, PFCS 2 mm, and PFCS 3 mm). The distance between centres of drilled holes is 10 × 10 mm that are applied by a CNC machine. To prepare the test samples, two face sheets and a drilled foam core are placed on the flat glass table. A fabrication setup with air bubble-free resin vacuum infusion was used. A plastic bagging film covers all the setup. The polyester resin (Epicote 2175, Hexion, Bangkok, Thailand) from a resin tank is transferred to the fabrication medium and after filling up the setup the additional resin will be trapped in a resin chamber. For PFCS samples the resin should fill all the holes. For each test sample, five specimens were fabricated regarding ±1% weight error. Figure 1 shows a schematic view of the fabrication setup for FCS and PFCS panels [3].

**Figure 2:**
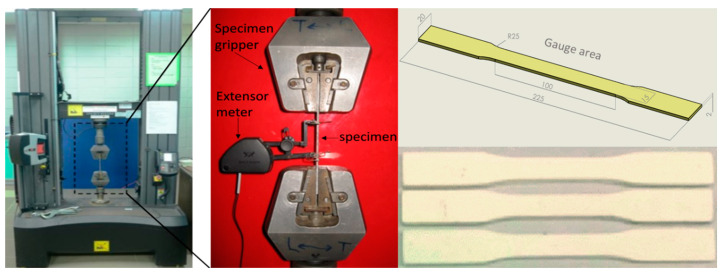
Tensile testing machine and tensile test samples to extract the material properties of resin pins.

**Figure 3:**
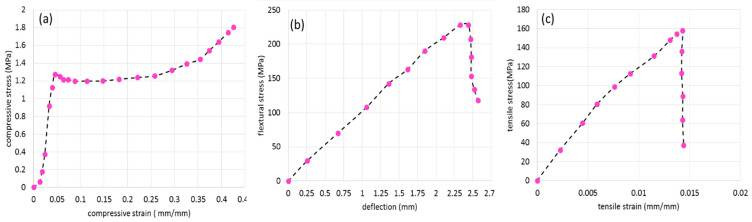
(**a**) The compressive stress–deflection response of polyurethane foam core, (**b**) the flexure stress–flexure deflection curves of glass/polyester laminate, (**c**) The stress-strain curves of glass/polyester laminate [9].

The samples of the core and face sheets as the constituents of the sandwich panel, were fabricated following the ASTM D3039 method and used for mechanical characterizations to obtain the properties and mechanical responses. In order to extract the material properties of polyester pins the dog bone shape samples were fabricated and based on ASTM standard, were tested as shown in Figure 2. The same size of tensile test samples was used for material testing of face sheet samples. The results of the tensile test for the face sheet are shown in Figure 3c.

The results of the stress-strain curve for core and face sheets are shown in Figure 3. The graphs show the average value of each test between five samples. Figure 3a shows the true compressive stress-strain curve for the polyurethane foam. Figure 3b presents the bending stress-deflection curves of three-point bending tests for glass/polyester laminate. Figure 3c shows the tensile stress-strain curve for glass/polyester laminate. The material properties of different materials are shown in Table 1. The material properties can be used for FE simulation.

**Figure 4:**
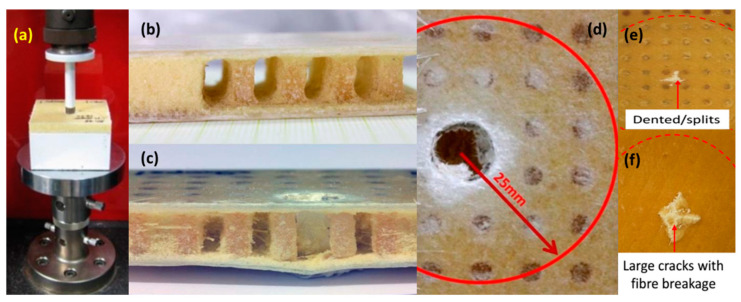
(**a**) Indentation test of foam core composite sandwich panel, (**b**) PFCS panel (foam partially removed to show pins), and the failure modes of PFCS panels (**c**) side view, and (**d**) top view (**e**) failure of back face for PFCS and (**f**) failure of back face sheet of FCS [31].

According to ASTM standard D6264M, quasi-static tests and low-velocity impact tests on different composite sandwich panels (FCS and PFCS) were conducted. A universal tensile testing machine (UTM, Instron, Norwood, MA, USA) was used to carry out indentation tests with different crosshead velocities (1, 10, 100, and 500 mm/min) (see Figure 4a). In order to verify the repeatability of the results, all tests were repeated five times. Figure 4b shows the partially removed foam from PFCS to represent the polyester pins before the test. Figure 4c shows the crushed pins at the end of the test under 100 mm/min loading rate. From Figure 4c it can be seen that the debonding occurs only between the bottom face sheet and pins. Moreover, only pins near to penetrated region were separated. Figure 4c,d show the penetrated area for a fully damaged structure under 500 mm/min loading rate. From Figure 4d can be observed that only the pins in the circular area meet the crack initiation. Near the penetrated area the failure of pins can be detected. Figure 4e,f compare two failed back face sheets of PFCS and FCS, respectively. It can be seen that the sample without pins has a large cracked area with fibre breakage however, the PFCS sample has a smaller failure area when both samples were subjected to the same loading condition.

## 3. Finite Element Model and Simulation

**Figure 5:**
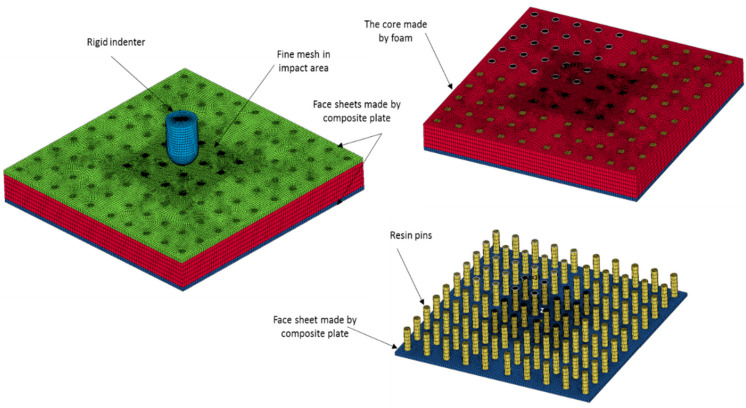
Presentation of FE model and detailed parts.

In order to study the behaviour of sandwich panels under different loading conditions a 3D FE model is generated in the LS-DYNA commercial software [32,33]. To create the FE model and appropriate element the mesh quality cannot be achieved directly. Therefore, a 3D CAD model was generated, and through the HYPERMESH software, the FE model was generated [34]. To reduce the computational time and simplification of the model a quarter of the model was generated. Figure 5 shows the meshed PFCS panel with detailed parts for FE simulation.

**Figure 6:**
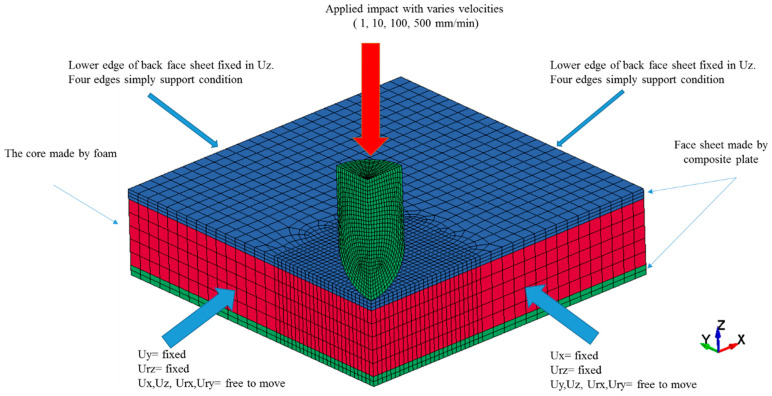
Boundary conditions and applied load to a quarter FE model.

The connectivity between pins and face sheets and also between core and face sheets is an important matter that was taken into account. By different geometrical criteria such as aspect ratio, skew angle, the minimum and maximum size of elements the quality of mesh was examined, and after that by using the Lagrange method the mesh convergence study was implemented [35]. The Tie-break interface model was applied between face sheets and core and pins interface [36]. A tie model was used to make perfect bonding between the foam hole’s surface and the outer surfaces of pins. The material properties of the resin, based on the physical properties of polyester resin were implemented in a NULL material model. To model the dry resin a null material card needs density, the module of elasticity, Poisson’s ratio, and fracture stress and strain as mentioned in Table 1. Based on the experimental test the tensile, shear, and compressive strength of the glass fibre/polyester face sheets were imported to the MAT_COMPOSITE_DAMAGE model (see Figure 3b,c). The properties of foam were implemented as a compressive stress-strain curve to the MAT_CRUSHABLE_FOAM material card (see Figure 3a). The material properties of foam core and face sheets were considered rate-dependent materials. Due to the lack of information about the rate dependency of polyester resin only the quasi-static mechanical properties of pins were considered. The element deletion criteria in this study followed the Table 1 fracture strain for each material. The impactor was considered as the rigid body and a surface-to-surface contact model with 0.05 friction coefficient was applied to the model. Four different FE models based on different categories (FCS, PFCS 1 mm, PFCS 2 mm, and PFCS 3 mm) were generated, and each model was simulated with four different loading rates (1, 10, 100, and 500 mm/min). The boundary conditions and applied load of a quarter FE model are depicted in Figure 6.

**Figure 7:**
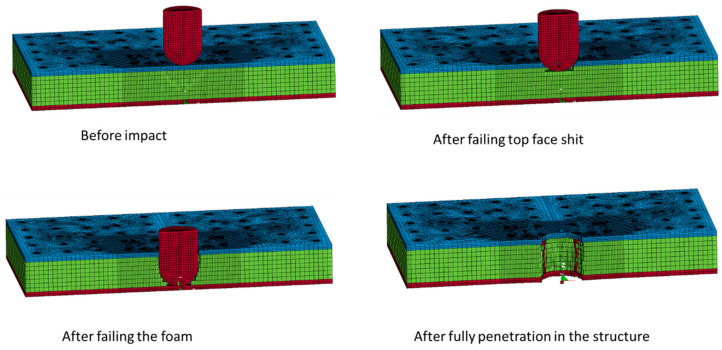
Failure process of a half cross-section of a 3 mm PFCS panel subjected to 500 mm/min over the time.

Figure 7 shows chronologically the graphical results of a half cross-section of a 3 mm PFCS panel subjected to 500 mm/min. It can be seen that during the test how different layers eroded and indenter penetrates in the sandwich structure. The bonding behaviour of different interfaces is close to experimental observation and at the end of the test, the maximum permanent deflection is related to the penetration area’s sounding pins.

## 4. Design of Experiment Analysis

Once the FE model was validated through experiments, the model was extended to find a better understanding of different factors affecting the structural integrity of the PFCS panel [2,37]. To study the data and evaluate the effective variables affecting sandwich panel response the DOE method was applied in this study. The half-central composition regarding the RSM response surface method was considered for the DOE method. The reason to choose half-central composition was to reduce the number of runs. In addition, the RSM was selected to generate the quadratic response surface in order to achieve more accurate results.

Firstly, in a DOE method, one has to define the main influencing factors, which are those affecting the structural dynamic responses. The next step of the design of experiments. In this study, two levels have been taken into account for three numerical factors (pin size, thickness of face-sheets, and impact velocity). One categorical factor (the size of the impactor) is considered. The diameter of the indenter was considered 25 and 30 mm.

The upper and lower thresholds were considered based on physical limitations. The value of the un-coded parameters as the upper and lower limit of each factor can be seen in Table 2.

In this study two different responses, i.e., energy absorption and maximum peak load were considered. In this case, the results of a numerical simulation have no fluctuation and the results repeatability can be achieved for every single simulation. Therefore, no replication has been considered. Considering half central composition (quadratic) method with three continuous factors, one categorical factor two different responses and one centre point generate 24 randomized trials for DOE analysis. After finishing every 24 trails, the statistical analysis will be implemented on the extracted data. Furthermore, the ANOVA analysis was performed to analyze the data statistically.

## 5. Results and Discussion

### 5.1. Experimental Results

**Figure 8:**
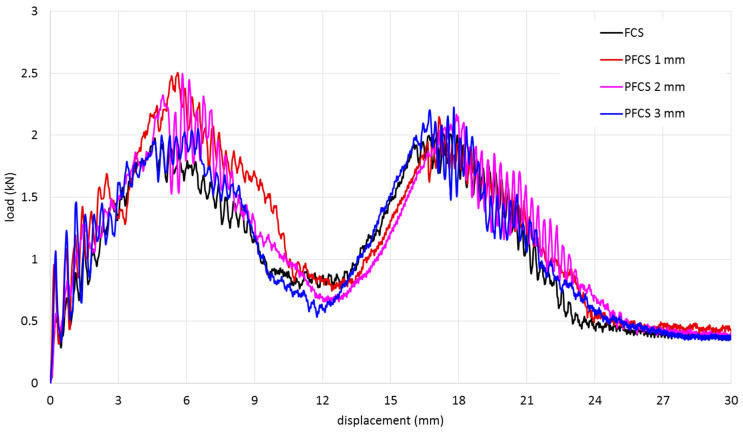
Comparison between experimental results of FCS and PFCS subjected to 500 mm/min.

The experimental results of FCS and PFCS subjected to 500 mm/min are shown in Figure 8. The maximum peak load is related to the case PFCS-2 mm. the weakest panel in this study was FCS panels. Based on the fractured area observation (Figure 4) it can be said that increasing the size of pins will affect the bonding failure significantly. The results of all rates (1–500 mm/min) were compared and they follow the same trend. Using the pined sandwich structure can increase the energy absorption of a panel up to 20% however the weight of the structure can be increased slightly. For instance, in the case reported in Figure 8 the weights of each panel were 26, 31, 36, 41 gr for FCS, PFCS 1 mm, PFCS 2 mm, and PFCS 3 mm respectively. However, calculation of specific energy absorption proves that increasing the pins increase the weight of the structure infinitesimal whereas, the energy absorption of the structure was increased significantly.

### 5.2. Validation Procedure

After finishing the simulations the balance of energy for each simulation should be performed. This procedure can be a good tool to verify the numerical simulation for a dynamic problem. The expression of energy balance for a FE model can be written as follows [29,38]:

External Work = Kinetic Energy + Internal Energy + Friction Dissipation − Total Energy(1)

**Figure 9:**
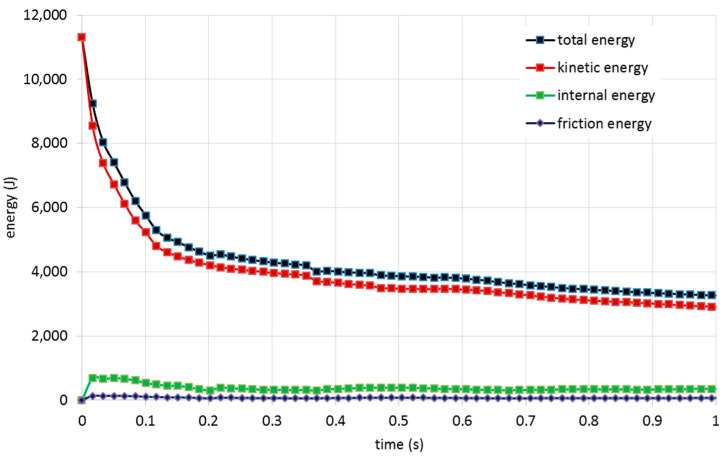
Energy balance representation for 3 mm PFCS panels subjected to 500 mm/min.

Figure 9 shows the balance of energy for the case PFCS 3 mm panels under 500 mm/min loading rate. It can be seen that the balance of energy based on Equation (1) has been satisfied and the error is less than 0.1%. It means that the numerical solution was performed perfectly and there is no noise and distortion can be detected in FE simulation. The quality of the mesh, applying correct contact and interface modelling, and correct boundary constraints have the main role to obtain realistic energy balance. The balance of energy for all cases was analyzed and the numerical validity of all cases has been verified.

**Figure 10:**
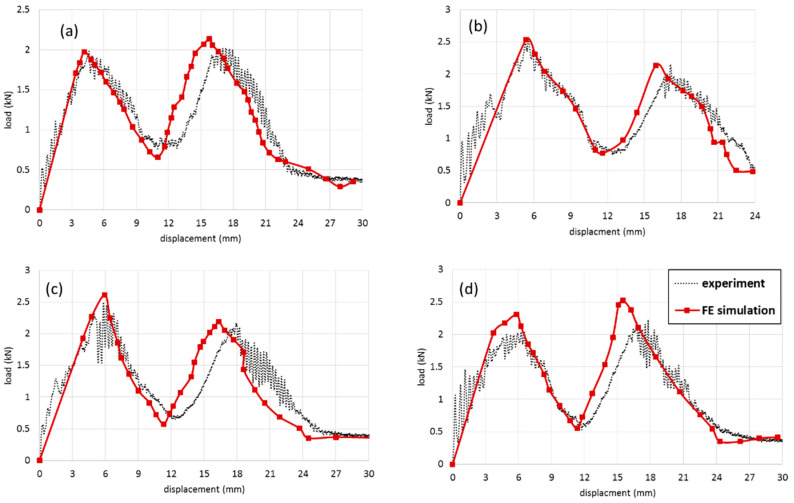
Comparison between experimental results and FE simulation for (**a**) FCS, (**b**) 1 mm PFCS, (**c**) 2 mm PFCS, (**d**) 3 mm PFCS panels subjected to 500 mm/min.

The results of FE simulations compared with experimental results and the validated results were considered as the baseline for further study. Figure 10 shows the comparison between FE and experimental results. The maximum error for all velocity cases was 500 mm/min that the error was less than 6%. Figure 10a shows the load-displacement curve of the FCS panel. Calculation of the area under the curve gives energy absorption of the sandwich structure. The first peak load is related to the stiffness of the upper face sheet and the second one is for the bottom face sheet. In all cases, the first and second peak loads slightly have the same value. It means that during the penetration process, the debonding failure does not occur and the energy for fracture of the first face sheet and second face sheet are close to each other. With this property, the full capacity of impact resistance of the sandwich panel can be achieved.

Figure 10b–d represent the load-displacement curves for PFCS 1, 2, and 3 mm panels, respectively. Comparison between the results of the FCS panel and PFCS show that using pin reinforced in the sandwich panel can increase the peak load and energy absorption of a panel up to 20%.

### 5.3. Effect of Different Loading Rates

**Figure 11:**
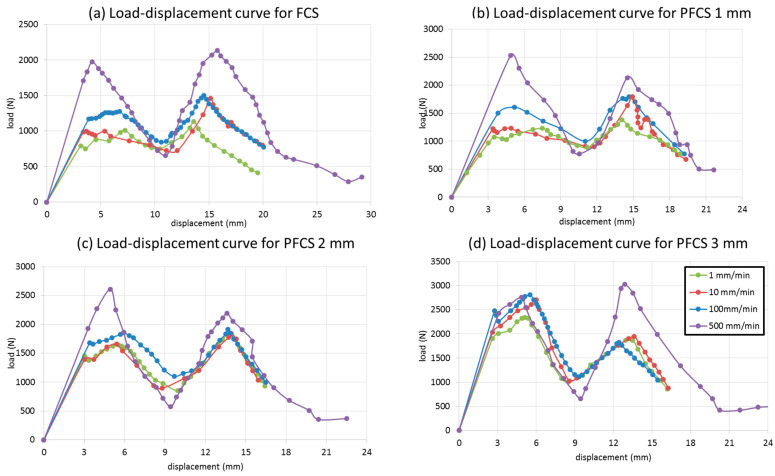
FE results for (**a**) FCS, (**b**) 1 mm PFCS, (**c**) 2 mm PFCS, (**d**) 3 mm PFCS panels under different loading rates.

Figure 11 shows the results of FE simulation for (a) FCS, (b) 1 mm PFCS, (c) 2 mm PFCS and (d) 3 mm PFCS panels, under different loading rates. The results show that the structures’ behaviour has the same trend below 100 mm/min. however, by increasing the velocity up to 500 mm/min the structural behaviour has different responses. It can be said the sandwich structure has rate-dependent behaviour and by increasing the impact velocity the peak load will be increased gradually. However, all the tests were performed in quasi-static (1 and 10 mm/min) and low-velocity impact (100 and 500 mm/min) regions. Comparison between the size of pins from Figure 11b–d show that there is a significant improvement can be obtained by increasing the pin size. Comparing Figure 11a and Figure 10d shows a 25% peak load enhancement that significantly increases the impact resistance of a pin-reinforced sandwich plate. The results were compared with experimental finding and the maximum error between all cases were less than 6%. The accuracy of the results leads to develop the FE model for DOE analysis.

### 5.4. Failure Modes on FCS and PFCS Panels

**Figure 12:**
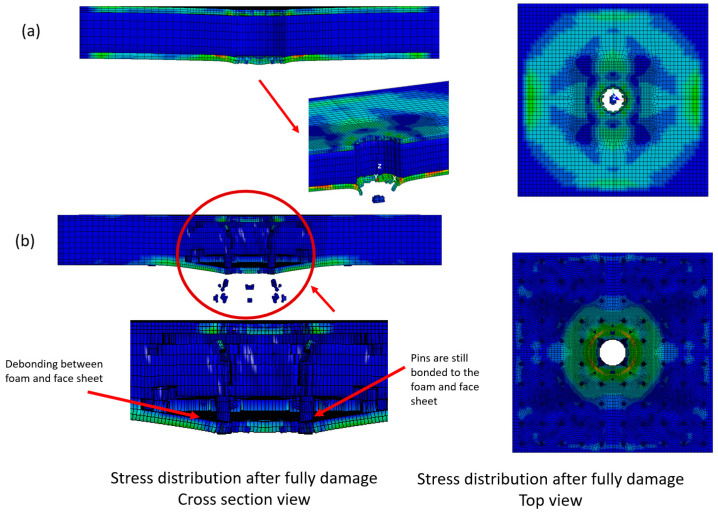
Stress distribution and failure observation of (**a**) FCS, (**b**) 3 mm PFCS panels subjected to 500 mm/min.

Figure 12 illustrates the stress distribution of on the (a) FCS, (b) 3 mm PFCS under 500 mm/min. the stress distribution of the FCS panel shows that the stress propagated on the structure after failing the structure. On the other hand, the stress contour of the PFCS panel shows a uniform distribution around the impact area. It can be interpreted that using pin reinforced can control stress propagation in a specific location as well as increase of the bending strength. Figure 12b shows the debonding failure between the lower face sheet and foam core however, the boding between pins and face sheet and foam core remains. Referring to Figure 4c the results of FE simulations and experimental testing have a good agreement in visual detection as well. The difference between failure modes of FCS and PFCS panels is that in FCS panels after the failure of matrix and fibres of the first face sheet there is no bending resistance through-thickness and the next face sheet should resist against the load individually. However, in PFCS panels after test initiation, a portion of bending moment is transferred to the pins and the breakage load should be increased to fail the first face sheet. Regarding the next face-sheet it can also be said that the pin holds the face sheet until the end of the test to avoid more debonding failure. It helps to increase the energy absorption of PFCS panels up to 20% as mentioned previously.

### 5.5. ANOVA Analysis and Verification Test

In order to assess the effectiveness of each factor a design matrix based on the randomized RSM method was prepared. The first factor was the thickness of the face sheet varies between 1 to 2 mm, the second factor was pin size varies between 0 to 5 mm and the third continuous factor was impact velocity between 1 to 500 mm/min. the only categorical factor was using two diameters of hemispherical indenter by 25 and 30 mm. regarding the applied threshold a design matrix by 24 random experiments was designed and all the tests based on the matrix were conducted through the FE method. The results were extracted from LS-DYNA and transferred to design Expert software as the responses of the DOE analysis. The analysis of variance was performed with two different responses (energy absorption and maximum peak load).

According to ANOVA analysis in Table 3 and Table 4, to investigate both responses; energy absorption and maximum peak load, all main factors are significant with a *p*-value of less than 0.06 for 94% confidence. The R-squared value shows how accurate the model is in predicting the response values. Also, the measurement of the amount of variation around the mean. An R-squared value close to 1.0 is desirable. The value of R-square for energy absorption response is 0.992 and for peak load was 0.934, which shows the high accuracy of the model. Moreover, the difference between the Predicted R-square of 0.92 and the adjusted R-square of 0.86 is less than 0.1 which is desirable. The signal-to-noise ratio is illustrated by the adequate precision that compares the range of the predicted value at the design points to the average prediction error. A desirable ratio should be greater than 4, while this value for the first response is higher than 45 and 17, respectively, which shows the high confidence of this model.

**Figure 13:**
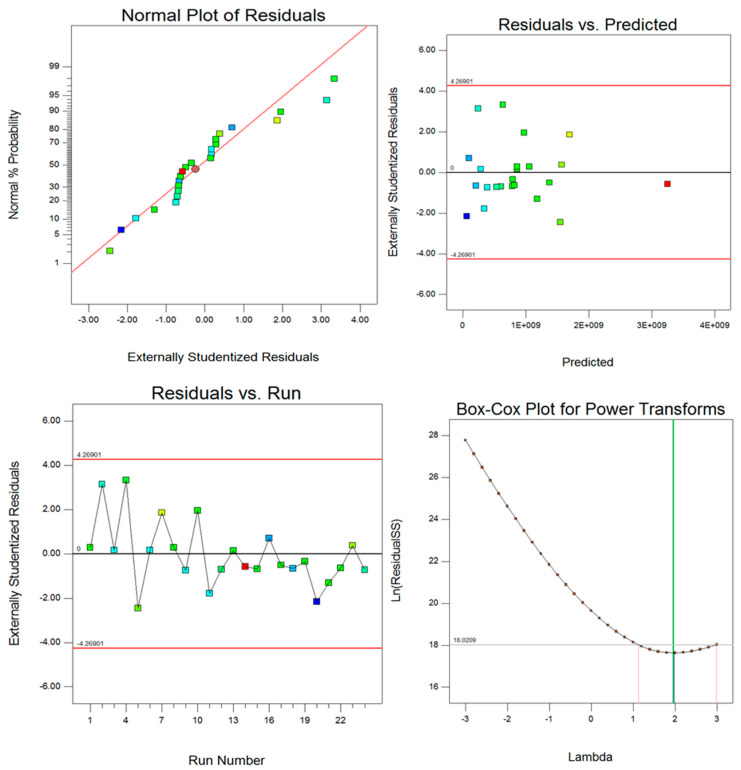
Post ANOVA verification tools for the prediction of energy absorption.

**Figure 14:**
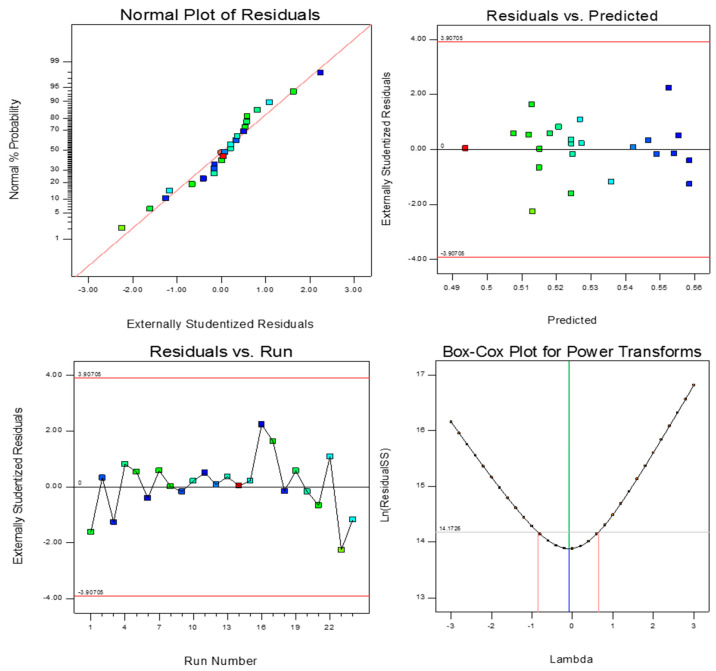
Post ANOVA verification tools for the prediction of maximum peak load.

To evaluate the accuracy of the result four main tools such as normal distribution, residual error, run vs. predicted error, and Box-Cox transformation were used. Implementation of these four criteria verifies the accuracy of the results. Figure 13 and Figure 14 show the normal distribution, residual error, run vs. predicted error, and Box-Cox transformation for two different responses (energy absorption and maximum peak load). From the following figures, it can be interpreted that the selected models for both responses were acceptable and the normal distribution of data proves verification of the screening method for all factors. It is worth mentioning that in both response’s ANOVA analyses, the most effective parameter was the pin size.

Post ANOVA verification leads to the generation of an empirical model to predict each response in the range span of every defined factor in Table 2. Equations (2) and (3) are the un-coded proposed models to predict the energy absorption and peak load for FCS and PFCS panels.

*Energy = 34600.53 + 2547.94 ∗ A + 12202.69 ∗ B + 6064.65 ∗ C + 4128.51 ∗ D + 1014.97 ∗ AB + 1050.6 ∗ AC − 276.86 ∗ AD − 251.35 ∗ BC + 1175.02 ∗ BD + 959.71 ∗ CD − 2378.36 ∗ A^2^ + 6578.85 ∗ B^2^ − 2941.27 ∗ C^2^* (2)

*Ln (max load) = 8.22 + 0.09 ∗ A + 0.46 ∗ B + 0.16 ∗ C + 0.13 ∗ D − 0.034 ∗ AB − 1.99 × 10^−3^ ∗ AC − 4.02 × 10^−4^ ∗ AD − 0.041 ∗ BC + 0.016 ∗ BD + 0.026 ∗ CD − 0.054 ∗ A^2^ + 0.052 ∗ B^2^ − 0.21 ∗ C^2^* (3)

**Figure 15:**
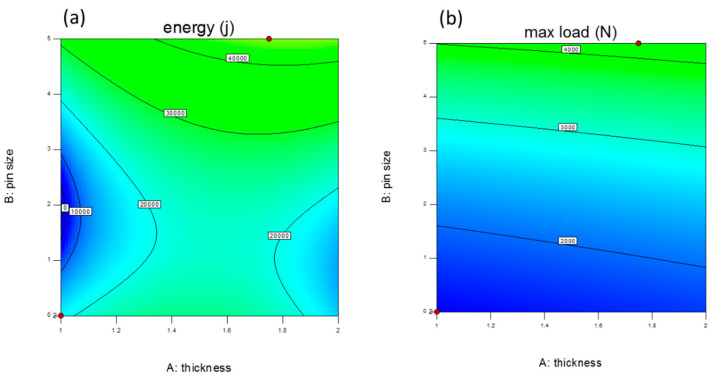
Response prediction plot for (**a**) Energy absorption and (**b**) maximum peak load.

Figure 15 represents the counter of prediction value for responses of DOE analysis when the velocity is constant at 100 mm/min. The value of energy absorption by FCS and PFCS panels subjected to a constant loading can be predicted by this contour graph. Based on the graph the best design can be considered with 1.8 mm face sheet thickness and 5 mm pins diameter. However, increasing the pin size after 4.5 mm doesn’t increase the value of energy absorption and the failure initiate earlier when the pin is 5 mm. the accuracy of the model has been examined by repeating the experimental test and the difference between the statistical model and experimental finding was less than 9%. Therefore, using an empirical model can be useful to accelerate the prediction of FCS and PFCS panels with different design specifications.

## 6. Conclusions

Numerical simulation and experimental test of low-velocity indentation test were performed on FCS and PFCS panels. By reinforcing the foam core of the FCS panel with cylindrical polymer pins, the indentation strength of the sandwich had improved significantly. A 3D FE model was generated and based on the experimental condition the simulations were performed. The numerical model was validated through experimental results with desirable accuracy. The results show that the size of pins has a significant effect on the performance of PFCS panels. The effect of loading rate is the second significant parameter in this study. To find the optimum condition an RSM DOE model was generated and after 24 random runs, an empirical model was obtained to predict energy absorption and the maximum peak load of FCS and PFCS panels. The weight of PFCS panels is higher than FCS panels however, using PFCS panels can increase the energy absorption of the structure up to 20%. The proposed methodology leads to the finding of the optimum sandwich panel instead of increasing the thickness of panels by conventional methods.

## Figures and Tables

**Table 1:** Mechanical properties of material contents of PFCS panels [9].

Material	ρ (kg/m^3^)	E (Gpa)	ν	$\varepsilon_{f}$	$\sigma_{f}\;(\mathrm{MPa})$
PU foam (core)	139.1	0.125	0.05	0.025	1.293
Polyester resin (pins)	1200	4	0.4	0.65	80
Glass fiber/polyester (face sheets)	1150	15.75	0.22	0.0136	154.1

**Table 2:** The specifications of the DOE design matrix.

Study Type	Response Surface Randomized									
Design Type	I-Optimal		Runs	24						
Design Model	Quadratic									
**Factor**	**Name**	**Units**	**Type**	**Subtype**	**Minimum**	**Maximum**	**Coded**	**Values**	**Mean**	**Std. Dev.**
A	thickness		Numeric	Continuous	1	2	FALSE	1.000 = 2	1.48958	0.413621
B	pin size		Numeric	Continuous	0	5	FALSE	1.000 = 5	2.27083	1.96147
C	velocity		Numeric	Continuous	1	500	FALSE	1.000 = 500	173.917	217.173
D	impactor		Categoric	Nominal	1	2			Levels:	2
**Response**	**Name**	**Units**	**Total run**	**Analysis**	**Minimum**	**Maximum**	**Mean**	**Std. Dev.**	**Ratio**	**Trans**
R1	energy	j	24	Polynomial	3706.69	63,637.6	29,936	12,917.1	17.1683	Power
R2	max load	N	24	Polynomial	3090.95	6774.3	3981	1447.55	22.5097	None

**Table 3:** ANOVA analysis for Energy absorption as the first response of DOE method.

Response	1	Energy Absorption			
Transform:	Power	Lambda:	1.98	Constant:	0
	Sum of Squares	df	Mean	*F* Value	*p*-Value Prob > F
Source			Square		
**Model**	1.12 × 10^19^	13	8.62 × 10^17^	102.49	<0.0001
**A-thickness**	2.84 × 10^17^	1	2.84 × 10^17^	33.72	0.0002
**B-pin size**	8.69 × 10^18^	1	8.69 × 10^18^	1033.12	<0.0001
**C-velocity**	8.53 × 10^17^	1	8.53 × 10^17^	101.4	<0.0001
**D-impactor**	5.82 × 10^17^	1	5.82 × 10^17^	69.12	<0.0001
**Residual**	8.41 × 10^16^	10	8.41 × 10^15^		
**Std. Dev.**	9.17 × 10^7^		R-Squared	0.9926	
**Mean**	8.56 × 10^8^		Adj R-Squared	0.9829	
**C.V. %**	10.72		Pred R-Squared	0.8521	
**PRESS**	1.67 × 10^18^		Adeq Precision	45.475	

**Table 4:** ANOVA analysis for max peak load as the second response of DOE method.

Response	2	Max Peak Load			
Transform:	Power	Lambda:	−0.83	Constant:	0
	Sum of	df	Mean Square	*F* Value	*p*-Value Prob > F
Source	Squares				
**Model**	2.07 × 10^6^	10	2.07 × 10^5^	18.54	<0.0001
**A-thickness**	6.11 × 10^3^	1	6.11 × 10^3^	0.55	0.4723
**B-pin size**	1.76 × 10^6^	1	1.76 × 10^6^	158.14	<0.0001
**C-velocity**	4.18 × 10^4^	1	4.18 × 10^4^	3.75	0.0749
**D-impactor**	4.35 × 10^4^	1	4.35 × 10^4^	3.9	0.0698
**Residual**	1.45 × 10^5^	13	1.11 × 10^4^		
**Std. Dev.**	105.56		R-Squared	0.9345	
**Mean**	752.11		Adj R-Squared	0.884	
**C.V. %**	14.04		Pred R-Squared	0.8604	
**PRESS**	1.86 × 10^6^		Adeq Precision	17.124	

## Data Availability

The data presented in this study are available on request from the corresponding author.
